# Reflections on 25 hours a day at Ewha Womans University College of Medicine from August 2021 to January 2025: a dean’s farewell message

**DOI:** 10.12771/emj.2025.00045

**Published:** 2025-03-11

**Authors:** Eunhee Ha

**Affiliations:** Department of Environmental Medicine, Ewha Womans University College of Medicine, Seoul, Korea

Dear esteemed students, staff, alumnae, and faculty members of Ewha Womans University College of Medicine,

On August 1, 2021, when I assumed the significant responsibility as the 26th dean of the College of Medicine [1], I devoted myself as if each day provided 25 hours to advancing both Ewha Womans University College of Medicine and Ewha Medical Center. Over the past 3 years and 6 months, our college has achieved remarkable milestones in various fields amid the sweeping changes of the Fourth Industrial Revolution (Supplement 1). These accomplishments include promoting interdisciplinary research, pioneering advancements in medical education, enhancing school promotion, and expanding partnerships with external organizations as follows:

## Advancement of medical education to lead the future of the College of Medicine and communicate with the world

### Advancing the medical education system

We improved the quality of medical education by establishing the Ewha Medical Education Center (EMEC) [2]. We also maintained the “6-year accreditation” in medical education, underscoring the excellence of our programs.

### Expanding international exchange

We broadened our international collaborations with renowned universities such as the University of Tokyo and Stanford University by hosting joint international academic symposiums, reinforcing our position as a leading global women’s medical school.

### Strengthening student research capabilities

We introduced the Creative Research Challenge Course to encourage student participation in research through a structured program that begins in the second year of pre-medical studies, thereby contributing to the evolution of future medical education.

### Establishing extracurricular programs through the Green Ribbon Project

During students’ leaves of absence due to medical school quota issues starting in February 2024 [3], we supported students in planning their futures and nurturing their vision through the Green Ribbon Project. This initiative included self-directed learning programs via the future medical education platform, mentoring by senior students, student research programs, corporate internships, and various open lectures.

## Advancement of the College of Medicine through promoting research activity

### Strengthening support for graduate students

We established a new scholarship for incoming graduate students to nurture future talents and organized the Future Ready Research Festival to ignite research enthusiasm among both graduate and undergraduate students.

### Strengthening collaboration with external organizations

We cultivated an enabling research environment through sponsorship agreements with various institutions such as Seoul Clinical Laboratories, Seegene Medical Foundation, and the Cardiovascular Welfare & Research Institute. In addition, we inaugurated the Ewha-SCL Environmental Health Research Center to pioneer new research fields.

### Encouraging faculty research and motivation

We instituted the Ewha Womans University College of Medicine Academic Award for professors.

### The *Ewha Medical Journal*, a leading global academic journal

**Fig. 1.**
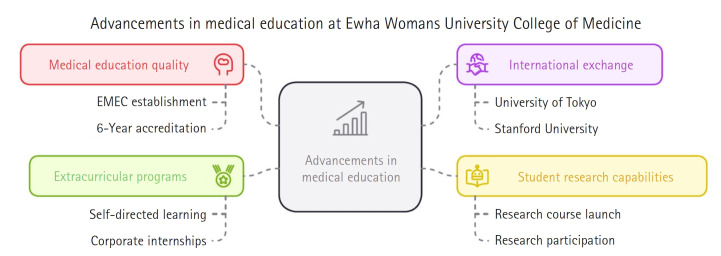
Diagram of the advancement of medical education to lead the future of the Ewha Womans University College of Medicine and communicate with the world. EMEC, Ewha Medical Education Center.

Key steps included inviting Dr. Sun Huh—a renowned expert in medical journal editing—as an editor beginning in September 2023; publishing a special issue that highlights current medical trends; and offering students opportunities to participate in the editorial process to secure the journal’s long-term growth starting in 2024 (Fig. 1).

## Strengthening school promotion and communication with society

### Enhancing medical school branding

We announced a school slogan, “Future Ready Ewha Medicine,” and launched a medical student ambassador group “EuiRang” to promote the school.

### Building various communication channels

We strengthened communication and networking with the alumnae association, students, and faculty to support student activities and increase students’ sense of belonging through mentor-mentee programs and various events.

### Fostering the Ewha Cutting-Edge Medi.Healthcare Cluster

**Fig. 2.**
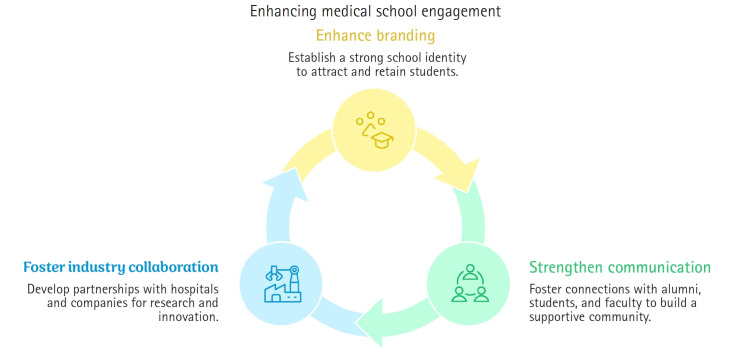
Diagram of steps taken to strengthen school promotion and enhance communication with society by the Ewha Womans University College of Medicine.

Through university–industry–hospital collaboration, we established a framework for research planning, performance management, and commercialization with companies in Magok and those affiliated with the Mokdong Hospital’s Industry-Academia Cooperation Center [4] (Fig. 2).

## Future vision

Ewha Womans University College of Medicine will continue to evolve as the leading institution for educating women physician-scientists and doctors in Korea. We will strive to cultivate future medical professionals equipped with the interdisciplinary thinking and practical skills required in the era of the Fourth Industrial Revolution. Our focus will be on investing in innovative research areas that shape the future of medicine, thereby contributing to the advancement of medical science. We will also fulfill our role as a medical school engaged with society by expanding our medical volunteer activities in collaboration with the local community and by developing educational programs that promote public health. This reflection will serve as a compass, commemorating the dean’s efforts in advancing our college and guiding the direction in which we must move forward.

## Sincere appreciation to the members and alumnae of Ewha Womans University College of Medicine

During my tenure as dean over the past 3 years and 6 months, our remarkable achievements have been possible solely due to the unwavering support and cooperation of our faculty, students, alumnae, and external partners. I would like to take this opportunity to express my deepest gratitude.

In particular, I am sincerely thankful to the members of the Ewha Womans University College of Medicine Executive Committee, including 4 vice deans, 6 directors, 15 deputy directors, 1 team head, and 27 Self-Evaluation Committee members for Accreditation and Evaluation of Medical Education, who devoted themselves to the significant progress of our institution. Their generous support for research initiatives, the creation of an innovative education system leading the future of medicine, and the enhanced promotional activities that elevated Ewha’s prestige have been decisive in propelling our college forward.

During my tenure as dean, I encountered many challenges and difficulties, yet we overcame them through the dedicated efforts and passion of the Ewha faculty, students, and alumnae, along with generous support and collaboration from external organizations. None of this would have been possible without the warm hearts and steadfast determination of the Ewha community. Thank you once again from the bottom of my heart.

Finally, as of February 2025, our students have not yet been able to return to medical school due to ongoing issues regarding the increase in medical school enrollment quotas in Korea [5]. I sincerely hope that there will be a swift return to normalcy in our medical education system.

## Supplementary materials

Supplementary files are available from Harvard Dataverse: https://doi.org/10.7910/DVN/X057VJ

Supplement 1. Yearly achievements during the author’s term as dean from August 2021 to January 26, 2025.
