# Supplementary material for: Reflections on 25 hours a day at Ewha Womans University College of Medicine from August 2021 to January 2025: a dean’s farewell message
Source: Ewha Med J. 2025 Mar 11;48(2):e20. doi: 10.12771/emj.2025.00045 (PMC12277507; doi:10.12771/emj.2025.00045)
Supplement: Supplementary file 1 — Supplement 1. Yearly achievements during the author’s term as dean from August 2021 to January 26, 2025. [file emj-2025-00045-Supplement-1.docx]

**Supplement 1.** Yearly achievements during the author’s term as dean from August 2021 to January 2025

Key initiatives and achievements by year from https://www.ewhamed.ac.kr

| **Year** | **Key initiatives and achievements** |
| --- | --- |
| Aug 2021– | - Inauguration of the 26th Dean of College of Medicine: Professor Eunhee Ha |
|  | - MOU signing with the Korea National Institute for Bioethics Policy |
|  | - Establishment of scholarships for incoming graduate students |
|  | - Research Fund Sponsorship Agreement with Seoul Clinical Laboratories |
|  | - Seegene Medical Foundation, College of Medicine and Ewha Medical Center Research Scholarship–Education Fund Sponsorship Agreement |
| 2022 | - Establishment of the 1st Future Ready Research Festival and awarded the Best Researcher Award for Students |
|  | - Establishment of the 1st Ewha Medical Academic Award for Professors |
|  | - Development of the public relations strategy of the College of Medicine: “Future Ready Ewha Medicine” |
|  | - Declaration of the College of Medicine’s slogan: “Future Ready Ewha Medicine” |
|  | - Launching the College of Medicine student public relations team “EuiRang” |
|  | - Cardiovascular Welfare & Research Institute donated funds for the development of College of Medicine: Ewha Medical Academic Award for Professors |
|  | - Opening of the Ewha-SCL Environmental Health Research Center |
|  | - Establishment of the Ewha Medical Education Center (EMEC) |
|  | - Acquiring ‘Maintained Accreditation’ in the interim evaluation of the 2022 Medical Education Evaluation and Accreditation ‘6-year accreditation’ |
| 2023 | - Reappointment of the 27th Dean of College of Medicine: Professor Eunhee Ha |
|  | - Holding the 2nd Future Ready Research Festival and awarded the Best Researcher Award for Students |
|  | - Holding the 2nd Ewha Medical Academic Award for Professors |
|  | - Co-Hosting the 1st Ewha Womans University College of Medicine & University of Tokyo College of Medicine Joint International Academic Symposium at Ewha Womans University |
|  | - Hosting Ewha Womans University College of Medicine & Stanford University Joint Seminar and Student Forum |
|  | - Launching Creative Research Challenge Course for student creative research challenge program |
|  | - MOU with Healthcare Knowledge Platform Weknew Inc. |
|  | - Opening the Ewha Global Health Research Center |
|  | - Implementing support projects for students’ excellent paper |
|  | - Appointing Professor Sun Huh, a renowned international expert in medical journal editing, as editor-in-chief; publishing a special issue highlighting current medical trends |
| 2024 | - Holding the 3rd Future Ready Research Festival and awarded the Best Researcher Award for Students |
|  | - Holding the 2nd Ewha Medical Academic Award for Professors |
|  | - Opening the Medical Artificial Intelligence Research Center |
|  | - MOU with AI MEDTECH company Waycen Inc. |
|  | - MOU with Mongolian National University of Medical Sciences |
|  | - International Cooperation with Korea Association of Health Promotion, Magok Biocluster Industry-Academic Cooperation, Joint Research MOU |
|  | - Acquiring ‘Maintained Accreditation’ in the interim evaluation of the 2024 Medical Education Evaluation and Accreditation ‘6-year accreditation’ |
|  | - Establishing Extracurricular Programs through the Green Ribbon Project |
| –Jan 25 | - Co-Hosting the 2nd Ewha Womans University College of Medicine & University of Tokyo College of Medicine Joint International Academic Symposium at University of Tokyo |
